# Transcriptomic gradients of the human cerebellum

**DOI:** 10.1162/imag_a_00494

**Published:** 2025-02-26

**Authors:** Leana King, Maedbh King, Zonglei Zhen, Richard B. Ivry, Kevin S. Weiner

**Affiliations:** Department of Neuroscience, University of California, Berkeley, CA, United States; Helen Wills Neuroscience Institute, University of California, Berkeley, CA, United States; McGovern Institute, Massachusetts Institute of Technology, Cambridge, MA, United States; Department of Psychology, University of California, Berkeley, CA, United States; Beijing Key Laboratory of Applied Experimental Psychology, Beijing Normal University, Beijing, China; Faculty of Psychology, Beijing Normal University, Department of Neuroscience, Beijing, China

**Keywords:** transcriptomics, cerebellum, neuroimaging, parcellation

## Abstract

In the past decade, there has been major interest in understanding the role of transcriptomics in the functional and anatomical layout of the human brain. To date, almost all of the work linking transcriptomics to human brain function and structure has been restricted to the cerebral cortex. The culmination of this work has identified transcriptomics as an important shared principle that can tie together function, structure, and gene expression. However, largely missing from this work is the subcortex—namely the cerebellum. Here, we investigate whether transcriptomics offer a link between function and structure in the human cerebellum, using gene expression data from post-mortem cerebella and multi-modal brain atlases. We find that transcriptomic gradients from a sparse subset of genes align with a macroanatomical, rather than a functional - parcellation of the cerebellum, and the transition of the main gradient occurs at the horizontal fissure for the group, as well as individual cerebella. Conversely, when filtering for cortex-specific genes, there is an alignment with continuous functional gradients of the cerebellum, but not discrete parcellated areas.

## 1 Introduction

Understanding how gene expression contributes to brain organization across spatial scales, and examining how these relationships contribute to cortical networks, cognition, and behavior (Arnatkevičiūtė et al., 2023;Arnatkevičiūtė, Fulcher, & Bellgrove, 2021;Arnatkevičiūtė, Fulcher, Oldham, et al., 2021;Deco et al., 2021;Shine et al., 2022) is a major topic in neuroscience. Previous studies (Arnatkevičiūtė et al., 2023;Arnatkevičiūtė, Fulcher, & Bellgrove, 2021;Arnatkevičiūtė, Fulcher, Oldham, et al., 2021;Deco et al., 2021;Shine et al., 2022) revealed that small groups of actively transcribed genes contribute to the formation of functional hierarchies and gradients across the human cerebral cortex (HCC), as well as the layout of functional maps within cortical areas (Arnatkevičiūtė et al., 2019;Burt et al., 2018;Gomez et al., 2018,^2021^;Hawrylycz et al., 2012;Miller et al., 2014) and the cytoarchitectonic arealization of the HCC (L. King & Weiner, 2024).

In parallel, there has been increased interest in understanding the transcriptomic landscape of the human cerebellum. The cerebellum has a distinct molecular signature as revealed in a genome-wide analysis of the human brain (Sjöstedt et al., 2020). Transcriptomics have proven valuable in examining the genetic architecture of the human cerebellum during early development (Aldinger et al., 2021;Haldipur et al., 2019,^2022^), complemented by*in vivo*human neuroimaging to delineate the microstructural properties of the developing cerebellum (Liu et al., 2022). These methods have yielded significant advances in our understanding of structural changes within the human cerebellum and provide valuable benchmarks for future research on atypical cerebellar development.

The human cerebellum’s anatomical organization is well established (Ashida et al., 2018;Cerminara et al., 2015;Larsell, 1947), and recent functional magnetic resonance imaging (fMRI) work has advanced our understanding of the cerebellum’s functional layout (Guell et al., 2018;M. King et al., 2019;Stoodley et al., 2012). However, the role of transcriptomics in bridging the cellular and macro-level layout of the whole human cerebellum remains unclear. Here, we aim to bridge this gap by utilizing open-source gene expression data from the Allen Human Brain Atlas (Arnatkevičiūtė et al., 2019;Hawrylycz et al., 2012;Miller et al., 2014) and human brain atlases acquired using either resting-state (Buckner et al., 2011;Yeo et al., 2011) or multi-modal task-based (M. King et al., 2019) fMRI data. In particular, we examined the relationship between transcriptomic gradients and anatomical and functional regions of the human cerebellum. To do so, we employed hierarchical clustering and classification algorithms to analyze gene expression patterns across six post-mortem human cerebella. Subsequently, we assessed whether these patterns aligned better with the human cerebellum’s anatomical or functional organization. Our findings revealed that transcriptomic gradients do not correspond to divisions based on discrete functional boundaries, as defined by fMRI. Instead, these gradients align with anatomical landmarks, namely lobules and fissures, of the human cerebellar cortex. At both the group and individual levels, we identified two distinct clusters along an anterior-to-posterior axis, dividing Crus I and Crus II at the horizontal fissure. However, when employing cortex-specific genes (Burt et al., 2018;Fagerberg et al., 2014;Genovese et al., 2016), we observed an alignment with functional gradients rather than discrete functional boundaries of the cerebellum.

## 2 Methods

### 2.1 Experimental model and subject details

Cortical and cerebellar gene samples were obtained from six postmortem human donors via the Allen Human Brain Atlas. Details about the donors and cause of death can be found at the following link:http://human.brain-map.org. To summarize, the donors were:

- Male, 39 years, African American
- Male, 24 years, African American
- Male, 55 years, Caucasian
- Female, 49 years, Hispanic
- Male, 31 years, Caucasian
- Male, 57 years, Caucasian

### 2.2 Method details

#### 2.2.1 Datasets

All of the data that are analyzed in this paper were obtained from freely available datasets that were approved by the ethics committee of each institution.

1) AHBA:http://brain-map.org/
2) Cerebellar atlases:www.diedrichsenlab.org
3) Cortical atlases:https://surfer.nmr.mgh.harvard.edu/

#### 2.2.2 Gene expression preprocessing

The gene expression data were obtained from the Allen Human Brain Atlas (AHBA).

DNA microarray analyses were used to map gene expression from broadly sampled tissue from six post-mortem human brains (Hawrylycz et al., 2012). There was significant variation in the sampling of the brain from the cerebellum. Samples were normalized to ensure within- and between-brain comparisons. Each sample was indexed by voxel coordinates (x, y, z) in MNI-152 space (seeFig. 1B). For each tissue sample, expression magnitude for 29,131 genes was recorded, and 93% of these genes were sampled by at least two probes. For more details regarding acquisition and normalization of the microarray data, please refer to:http://help.brain-map.org/display/humanbrain/documentation/.

**Fig. 1.**
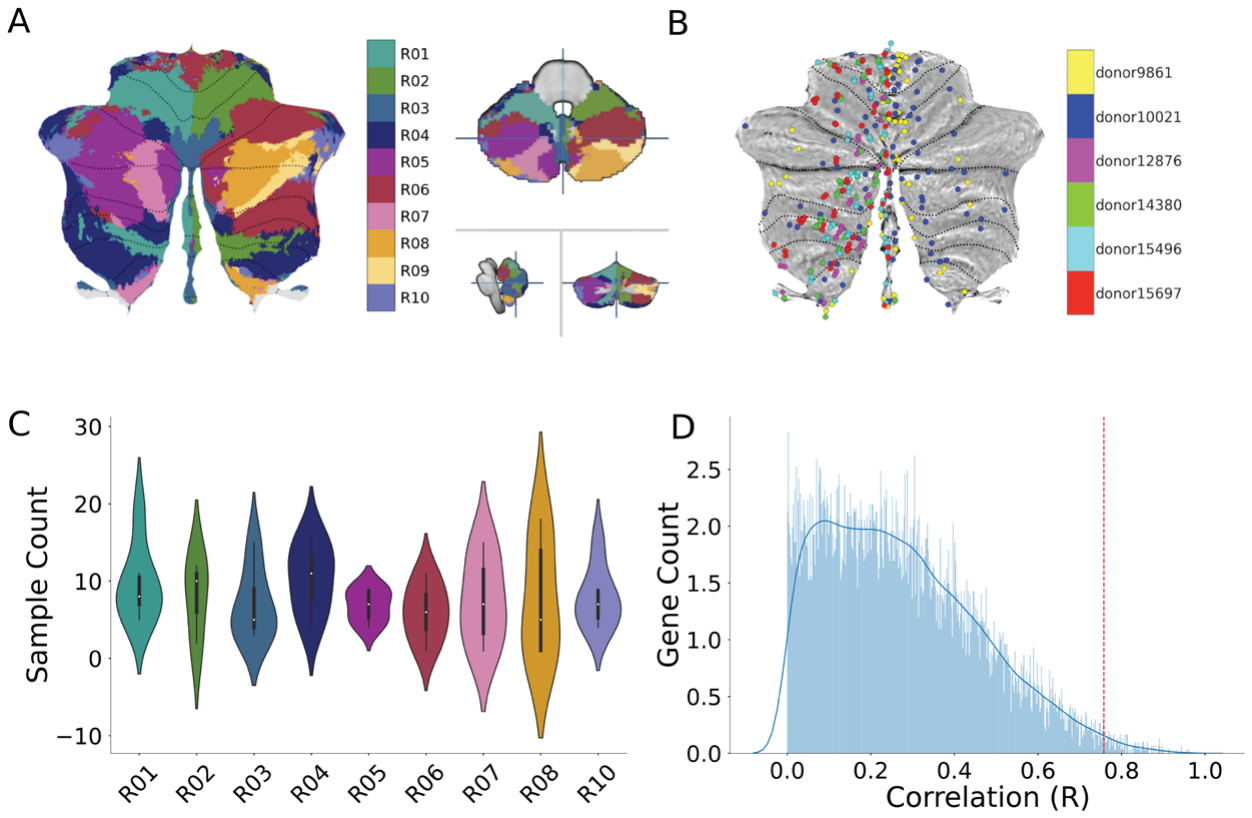
Relating transcriptomics and functional parcellations of the human cerebellum. (A) Ten functional networks (different colors) in the human cerebellum, derived from task-based fMRI data (M. King et al., 2019). Left: Flatmap representation of the networks (1-10). Right: Volume representation of horizontal (top), sagittal (bottom, left), and coronal (bottom, right) views. The flatmap is for visualization purposes only (Diedrichsen & Zotow, 2015); all analyses and statistics were conducted in the volume (Diedrichsen, 2006;Diedrichsen et al., 2009) (B) Individual donor samples are plotted in MNI coordinates and visualized on the flatmap. Each circle represents a sample, and each color denotes a donor. (C) Average sample counts across donors as a function of the 10 networks (R01-R10). No tissue samples overlapped with R09. (D) Histogram of gene stability. Gene selection was performed with a two-pronged, data-driven approach (section 2), which resulted in the selection of the top 1% (157) of genes, shown here as the area beyond the dashed red line.

Normalized microarray datasets were downloaded fromhttp://brain-map.org/, and the data were preprocessed using techniques outlined byArnatkevičiūtė et al. (2019), making use of the Abagen toolbox in python (Arnatkevičiūtė et al., 2019;Markello et al., 2021). Four steps were taken in the preprocessing pipeline: Re-annotating probes, selecting probes, matching tissue samples to each ROI, and normalization of expression data.

1. *Re-annotating probes:*Probes were re-annotated based on criteria outlined inArnatkevičiūtė et al. (2019). Probes that were not reliably matched to genes were discarded.
2. *Selecting probes:*Probes were selected on the basis of meeting two primary criteria. First, intensity-based filtering was applied to discard probes that did not exceed a specified level of background noise. The level (0.5) was set as the ratio of samples, across all donors, for which a probe had a signal above background noise. Second, as each gene was often sampled by multiple probes, a representative probe was selected. The representative probe was chosen by computing the Spearman correlation of microarray expression values for each probe across parcels for every donor-pair**.**Correlations were then averaged, and the probe with the highest correlation was retained. 15,662 genes remained after probe filtering and selection.
3. *Matching tissue samples to regions of interest (ROI):*Tissue samples were matched to regions of interest from a specified atlas (see*Atlas Templates*). This was done separately for each donor. A number of steps were taken to ensure that the sample was assigned to the correct ROI. When a sample fell directly within an ROI, it was assigned to that ROI and the matching process was terminated. When a sample did not fall directly within an ROI (i.e., was located on the boundary between two ROIs), the search space was expanded up to 2 mm to include nearby voxels. If the sample was located on the border of multiple ROIs, it was assigned to the closest ROI as determined by the distance between the sample and the ROI centroids. While this approach may result in some ROIs not having any assigned samples, it is a more exact method for assignment compared to one that forces each region to be assigned at least one sample. Once samples were assigned to an ROI, expression values for these samples were aggregated separately for each donor, resulting in an ROI x gene matrix for each donor.
4. *Normalization of expression data:*The expression data acquired from AHBA were normalized to account for expression-level differences across donors that might result from “batch effects”^2^. Despite this normalization, differences still remained in the data acquired from AHBA. To mitigate these differences, microarray expression data were normalized in two ways: 1) Each sample was normalized (z-scored) across all genes, and 2) each gene was normalized (z-scored) across all samples.

#### 2.2.3 Gene selection

Gene selection was performed using a two-pronged approach. First, the top 1% of genes that were most stable from the 10-network task-based functional parcellation were selected (Fig. 1A, D). The stability of gene expression was determined by computing split-half correlations across a split of the data (Group 1: Donors 9861, 10,021, and 12,876; Group 2: Donors 14,380, 15,496, and 15,697). Genes outside the 99^th^percentile of split-half correlations were discarded, resulting in a total of 157 genes. This thresholding approach minimizes false positives that might result from multiple comparisons (Gomez et al., 2019;L. King & Weiner, 2024). Second, to determine the optimal number of genes necessary to explain the variance across genetic expression patterns, a linear model was fit and validated on 80% of all donor samples (n = 256). The set of features that resulted in the lowest cross-validated error were considered the optimal feature set. A linear model was then fit using this feature set and evaluated on the held-out test set (n = 64). This analysis was done separately for each donor, grouped across fROIs and lROIs (lobular ROIs). The optimal number of features (i.e., genes), determined by the lowest cross-validated error, was 32 and 40 genes for the fROIs (test RMSE = 4.79, train RMSE = 4.69, KFold validation error = 5.28) and lROIs (test RMSE = 2.39, train RMSE = 1.73, KFold validation error = 1.92), respectively.

#### 2.2.4 Atlas templates

To test for a relationship between transcriptomic expression and the functional parcellation of the human cerebellum, we employed two parcellation schemes that have yielded comprehensive maps of the cerebellum in terms of functional regions of interest (fROIs). The first scheme is based on a task-based parcellation of the human cerebellar cortex (M. King et al., 2019). Relative to the horizontal fissure, each of the 10 functional regions in this map had an anterior and posterior segment, allowing us to create 20 unique regions. The rationale for this modification was to test whether functionally connected sub-regions (anterior and posterior portions of the same network) shared similar profiles of gene expression, despite being anatomically distinct.

The second scheme is based on resting-state fMRI data, with the cerebral cortex and cerebellum parcellated into either 7 corresponding networks or 17 corresponding networks (Buckner et al., 2011;Yeo et al., 2011). Here, too, we asked if functionally linked areas that are anatomically distinct have similar gene expression profiles. In this case, the anatomical separation is between the cortex and cerebellum.

To test for a relationship between transcriptomic expression and the lobular parcellation of the human cerebellum, gene samples were assigned to lobules (lROIs) based on a probabilistic atlas—the spatially unbiased infratentorial atlas (SUIT) (Diedrichsen, 2006;Diedrichsen et al., 2009).

In order to perform group analyses in a common reference space, the fROIs and lROIs were resampled into MNI coordinates.

#### 2.2.5 Extracting gene expression patterns

Gene expression patterns were examined relative to the three parcellations of the human cerebellum (task-based fROIs, resting-state fROIs, lobular lROIs) to test for functional- and/or anatomical-transcriptomic relationships. For these analyses, the same set of 157 genes was used. Samples were not obtained from four of the smallest fROIs (Fig. S1). SeeFigure 1Bfor full coverage of the samples across the cerebellar cortex. To conduct group analyses, samples from each atlas were averaged across subjects, resulting in a gene x ROI matrix for each atlas.

#### 2.2.6 Gene-set filtering

The top 157 genes, keeping with the same N from the original gene set, were selected from the ‘cortex-specific’ gene set (n = 2,413) reported by Burt and colleagues (Burt et al., 2018) using data fromGenovese et al. (2016)and Fagberg et al. (2014). This approach in AHBA has been established previously (Burt et al., 2018) and more recently used by our group relating transcriptomic variation to cytoarchitectonic boundaries (L. King & Weiner, 2024). This filtering approach is set to improve the sensitivity of the genes selected by eliminating noise from other non-cortical genes, such as sex- or cardiac-related genes. It is important to note that this gene set was derived from only cortical tissue and did not include any tissue from subcortical structures or the cerebellum. Thus, while the genes are ‘cortex-specific’, it is likely that many of these genes are also prominent in subcortical structures and the cerebellum as the present study highlights.

### 2.3 Quantification and statistical analysis

#### 2.3.1 Agglomerative hierarchical clustering

Gene expression patterns were input to an agglomerative hierarchical clustering algorithm for the modified task-based parcellation and lobular-based anatomical parcellation. Euclidean distances and the Ward method were used to cluster the data. The ordering of the fROIs and lROIs along the x- axis in the dendrogram is meaningful as the leaves are rooted and the clusters are not rotatable. To evaluate the significance of the dendrogram results for both of these atlases, we performed a bootstrap analysis with 10,000 iterations. On each bootstrap iteration, we randomly shuffled the gene expression profiles for each fROI and each lROI before submitting the profiles to hierarchical clustering. The Euclidean distance from the resulting ordered vector was then compared to the true ordered vector. The p-value was defined as the probability of observing a Euclidean distance of zero from the resulting 10,000 bootstrap samples.

#### 2.3.2 Representational structure

In order to examine the underlying structure of expression magnitude, gene expression patterns were organized and visualized in a raster plot in which each row corresponds to a gene and each column to a region-of-interest. The gene expression patterns were input to an agglomerative hierarchical clustering algorithm to determine the similarity in expression magnitude across genes. The procedure was the same as described in previous work (Gomez et al., 2019;L. King & Weiner, 2024), except that the leaves were gene samples, not regions-of-interest. The rows of the raster plot were reordered to align with the ordering of genes as determined by the dendrogram. We also visualized the representational structure with correlational matrices for each of the parcellations. Distance-dependent correlations were removed from the correlation matrices by regressing out the Euclidean distance between regions.

#### 2.3.3 Dimensionality reduction

Singular value decomposition was used to determine the most important components underlying gene expression patterns for both the functional and lobular atlases. This approach summarizes high-dimensional gene expression patterns along fewer dimensions, which allows for a visualization of additional sources of variance beyond the most dominant component*.*As seen in the visualization shown inFigure 3E, dimensionality reduction identifies a transcriptomic gradient, with a boundary at the horizontal fissure.

#### 2.3.4 Classification

To determine how well donor samples could be classified to functional and lobular regions, a logistic regression model was applied to the data. For the modified task-based parcellation, a binary classification approach was adopted to determine the classification of each of the 337 samples as being above or below the horizontal fissure, allowing a comparison with the true label for that sample based on its assignment in the modified task-based atlas. The logistic regression model was fit and validated on 80% of the samples and then evaluated on the remaining 20%. The accuracy scores were determined as the percentage of the prediction labels that were correctly assigned to the true labels and these scores were visualized in a confusion matrix (Fig. S2A). The F1 score was calculated by computing the harmonic mean of the precision and recall with a score of 1, indicating perfect precision and recall.

For the anatomical parcellation, a multi-class approach was adopted to evaluate classification of donor samples to lobules. In this approach, a binary problem was applied to multiple labels. The true label of each sample was given by its assignment to 1 of the 10 cerebellar lobules (I-X). A logistic regression model was fit and validated on 80% of the samples and evaluated on the remaining 20%. L2 regularization was applied to the model to prevent overfitting by penalizing large coefficients, improving the model’s generalization performance. The L-BFGS optimization algorithm was used to efficiently find the optimal model parameters that minimize the loss function while considering the regularization penalty. Otherwise, all default parameters were used. The accuracy scores were determined by computing the percentage of prediction labels that were correctly assigned to the true labels and these scores were visualized in a confusion matrix (Fig. S2B). A micro F1 score was calculated across all classes by counting the total true positives, false negatives, and false positives.

## 3 Results

### 3.1 Transcriptomic gradients do not align with a functional organization of the human cerebellum

**Fig. 2.**
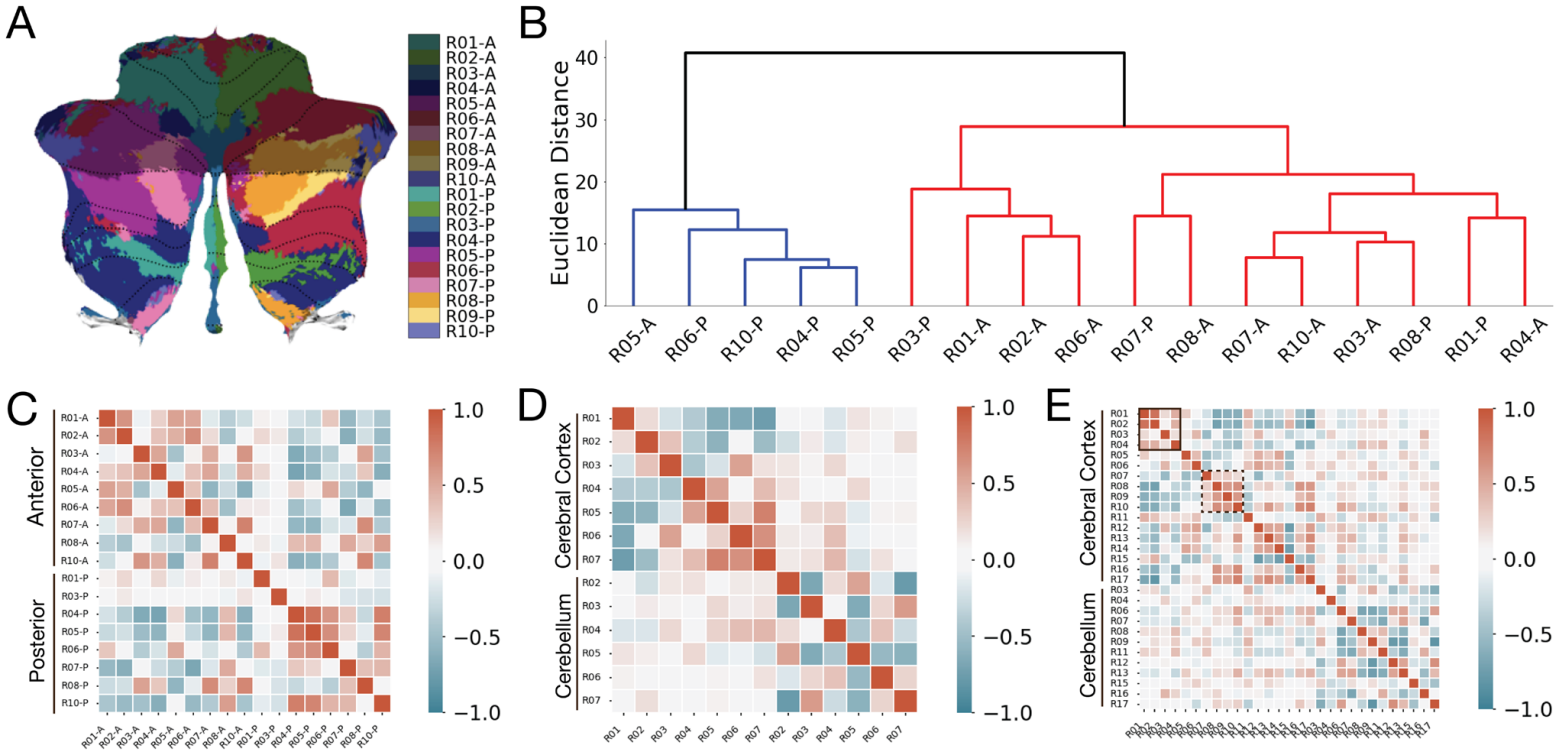
Transcriptomic gradients do not align with a functional parcellation of the human cerebellum. (A) Each of the 10 functional networks inFigure 1Awere subdivided into 20 functional regions of interest (fROIs) in which each network contained an anterior and posterior component; depicted as either darker or lighter shades. (B) Dendrogram reveals an algorithmic clustering of the fROIs in terms of transcriptomic gradients: At the highest level, regions are broadly clustered into regions that are either located above (red) or below (blue) the horizontal fissure. (C) Correlation matrix of gene expression for fROIs of the cerebellum. Distance-dependent correlations were removed by regressing out the Euclidean distance between fROIs as implemented in previous studies (Gomez et al., 2019,^2021^). (D, E) Correlation matrix of gene expression for fROIs of cortico-cerebellar networks using (D) a 7-network (seeFig. S1A) or (E) 17-network (seeFig. S1D) parcellation defined using resting-state fMRI. Red colors denote higher correlations, and green colors indicate lower correlations. As in C), distance-dependent correlations were removed by regressing out the Euclidean distance between fROIs. Black square in (E) denotes sensorimotor networks, and the dashed square denotes association networks from the Yeo 17-network parcellation.

To test whether transcriptomic gradients align with functional parcellations of the human cerebellum, we combined two open-source datasets: 1) gene expression data from the Allen Human Brain Atlas (AHBA) and 2) a functional parcellation of the cerebellum based on multi- modal task-based MDTB atlas (M. King et al., 2019) fMRI data. The MDTB atlas subdivides the cerebellum into 10 distinct networks (Fig. 1A), and each of these networks is spread across multiple lobular boundaries of the cerebellum. The functional networks can be separated into anterior and posterior functional regions of interest (fROIs;Fig. 2A), allowing us to ask the following question: Is gene expression more strongly associated with the anatomical or functional layout of the human cerebellum? If the former were true, clustering algorithms based on gene expression should situate anatomically proximal regions close to one another. Alternatively, these algorithms would situate functionally similar regions close to one another if the latter were true.

Cerebellar fROIs and gene samples were aligned to the MNI152 average brain, and gene samples were assigned to fROIs based on MNI coordinates (section 2). A twofold procedure was used to select genes from each fROI of the 10-network MDTB atlas. First, expression magnitude of the gene samples was correlated across two groups of donors, and the number of samples across both groups of donors was balanced. Second, to focus on genes that were the most reliably expressed across all 6 donors, we identified the top 1% of the most stable genes (Fig. 1D). Note that we also replicated our results with the top 5, 10, and 25% of stable genes (seeFig. S8). This procedure results in a gene (n = 157) by fROI (n = 20) matrix which was submitted to an agglomerative hierarchical clustering algorithm. Following previous methods in the cerebral cortex (Gomez et al., 2019), the fROIs were ordered according to the Euclidean distance of their expression profile from R01-A, the most anterior cerebellar fROI. Consequently, the dendrogram becomes “rooted” such that clusters at the lowest level cannot be rotated and the x-axis ordering in the resulting dendrogram meaningfully reflects inter-regional distances from R01-A.

At the highest level, the dendrogram clustered the fROIs along a major anatomical division within the cerebellum: The first cluster grouped regions above the horizontal fissure (anterior portions), while the second cluster grouped regions below the horizontal fissure (posterior portions). The exception to this separation of anterior and posterior ROIs occurred for R03P, R01P, and R08P, which are spatially contiguous regions that are more likely to belong to the same anatomical lobule, and thereby share the same profile of genetic expression (Fig. 2B).

To further quantify the clustering into anterior and posterior divisions, sample locations were classified using a binary logistic regression model. The likelihood of each sample being assigned to its true label (either anterior or posterior networks) was assessed by training the model on 80% of the samples and then testing the predictions on 20% of held out samples. Classification accuracy was 87% and 83% for samples located above the horizontal fissure and below the horizontal fissure, respectively (Fig. S2A). The F1 score, which computes a harmonic mean between precision and recall was.78. To test if our findings were specific to the anterior- posterior organization of the cerebellum, we repeated the analyses (using the same 157 genes) with both cortical and cerebellar fROIs from recent 7-network and 17-network resting-state atlases (Buckner et al., 2011). The correlation matrix of gene expression patterns across cortico-cerebellar fROIs revealed two distinct clusters (Fig. 2E): One cluster of non-zero (e.g., red or green shades) correlations among networks in the cerebral cortex and another cluster of non-zero correlations among networks in the cerebellum. The averages of all pairwise correlations for intracortical and intracerebellar networks were 0.35 and 0.34, respectively (Fig. S3B). Conversely, the average of all correlations between corresponding networks of the cerebral cortex and cerebellum was 0.17. Therefore, we conclude that the same genes that contribute to the anterior-posterior organization of the cerebellum also contribute to a clear transcriptomic dissociation between cerebral and cerebellar networks. Similarly, we observe that these genes also contribute to a differentiation of primary (networks 1-4) and association (networks 7-10) cortical networks (Fig. 2E). We found that the average pairwise correlations within primary networks (1-4, mean Pearson’s r = 0.38) and association networks (7-10, and r = 0.44) were positively correlated, while the primary and association networks were negatively correlated (mean Pearson’s r = -0.42) (Fig. S3C). These results suggest that the gene expression patterns underlying primary and association networks in the cerebral cortex are differentiated from one another, likely reflecting the differences in anatomy and/or functionality.

Specifically, while previous research supports a transcriptomic dissociation between cerebral and cerebellar cortices (Hawrylycz et al., 2012;Sjöstedt et al., 2020), here we show that a sparse set of genes parcellates i) the human cerebellum, ii) cerebral from cerebellar cortices, and iii) primary from association cortical networks.

### 3.2 Distinct transcriptomic gradients contribute to an anatomical division of the human cerebellar cortex occurring at the horizontal fissure

To further evaluate the relationship between genetic expression and the anatomical organization of the human cerebellum, we compared gene expression profiles using a lobular- based parcellation. Lobular ROIs (lROIs) were defined by the SUIT atlas, a spatially unbiased infratentorial template for the cerebellum (Diedrichsen, 2006;Diedrichsen et al., 2009). This atlas preserves the lobular nomenclature originally devised byLarsell (1947), spanning lobules I-IV in the anterior lobe, lobules V-IX in the posterior lobe, and lobule X in the flocculonodular lobe (Fig. S4). The same subset of genes extracted from the 10-region MDTB functional parcellation was used.

**Fig. 3.**
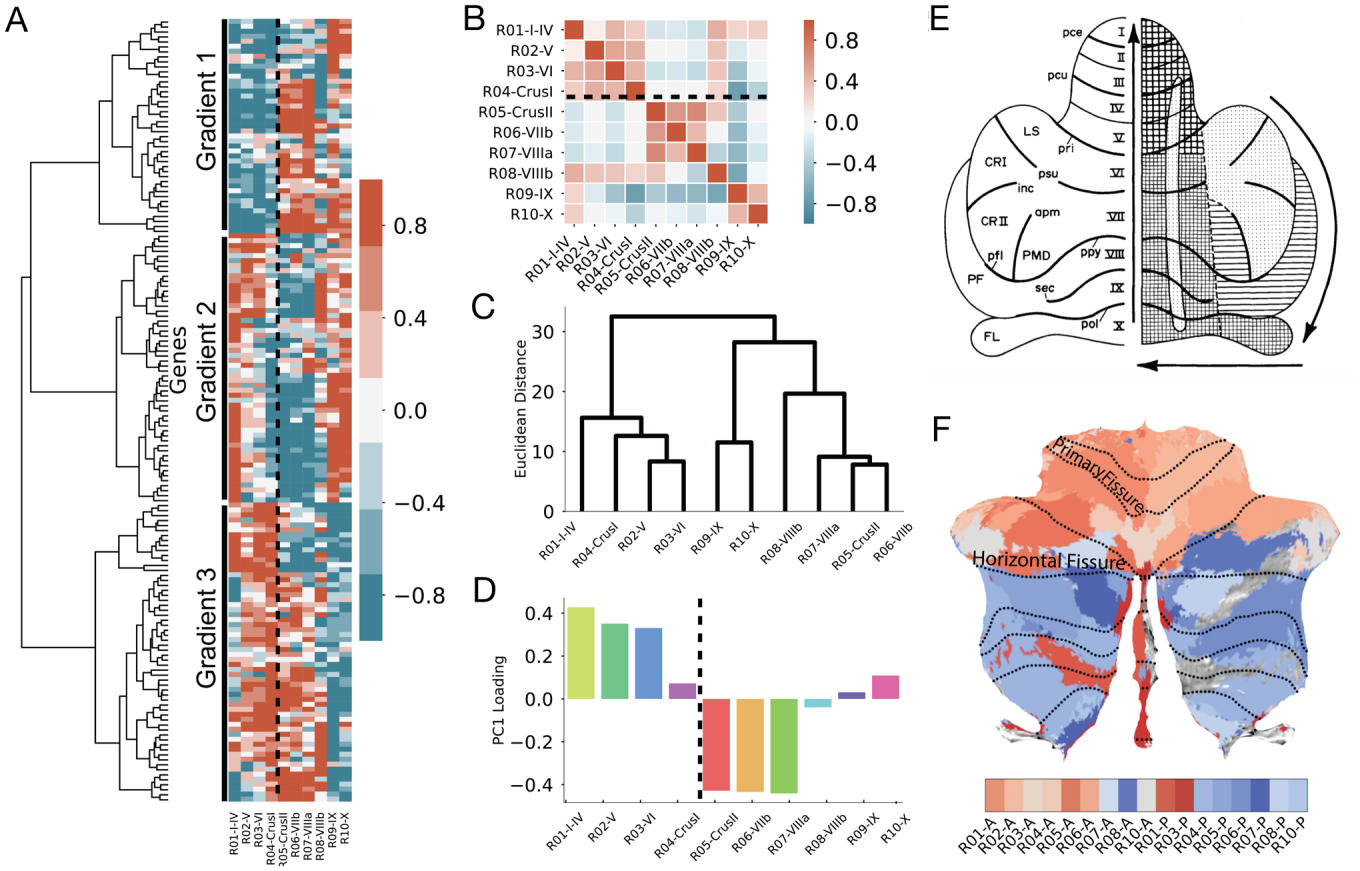
Distinct genetic gradients contribute to an anatomical division of the human cerebellar cortex. (A) Gene expression patterns are clustered into three gradients. Left column: An agglomerative hierarchical clustering algorithm was used to cluster the gene expression patterns. At the highest level, the genes predominantly cluster into three groups. Right column: Raster plot in which each column is a lobular region-of-interest (lROI) arranged according to its position along the anterior-to-posterior axis of lobular organization (R01-I-IV is on the left) and each row is a gene. The rows correspond with the dendrogram ordering such that each row of the matrix corresponds with each leaf of the dendrogram. Red indicates higher expression levels, while green indicates lower expression levels with expression levels normalized across all gene samples. The vertical dashed line denotes the boundary of the horizontal fissure. (B) Correlation matrix of gene expression for lROIs of the cerebellum. The horizontal dashed line denotes the boundary of the horizontal fissure. Distance-dependent correlations were removed by regressing out the Euclidean distance between lROIs. (C) Dendrogram reveals the algorithmic clustering of lROIs. (D) PC loading scores as a function of lobule for PC1. The first PC captures a crossover at the boundary of the horizontal fissure (vertical dashed line). Colors indicate the lobular labels from the SUIT parcellation shown inFigure S4. (E) Schematic illustration of the adult rat cerebellum fromAltman and Bayer (1985). Lobules and fissures are denoted on the left in accordance with Larsell’s nomenclature. The sequential order of Purkinje cell production is shown on the right. Similar to the present findings, the later stages occur along the posterior (light checkered) to anterior (heavy checkered) axis (depicted by black arrow). The horizontal fissure is denoted by ‘pri’. (F) Summary of the present findings on a flattened representation of the cerebellum. Transcriptomic differences between MDTB regions are highlighted via a color-gradient representing the Euclidean distance between regions taken from the hierarchical clustering inFigure 2B. The two colors, red and blue, represent the two top-level clusters inFigure 2B. Regions that are clustered together in the dendrogram are closer on the color spectrum than regions that are far apart with the deepest red and deepest blue representing regions on either end of the dendrogram.

Mirroring the previous analyses implemented for the fROIs, we used an agglomerative hierarchical clustering algorithm to cluster the gene expression patterns of the lROIs (seesection 2for details). The gene clusters are strikingly organized into three distinct gradients (Fig. 3A). The first gradient consists of genes that are highly expressed in lobules anterior to the horizontal fissure (lobule I-Crus I) and weakly expressed in lobules posterior to the horizontal fissure (Crus II - lobule X). The other two gradients are roughly mirror reflections of each other. One consists of a set of genes that are highly expressed in lobules V-VIIIb and weakly expressed in lobules I-IV, IX, and X; the other consists of a set of genes that are highly expressed in lobules I-IV, IX, and X and weakly expressed in lobules V-VIIIb.

To further evaluate the relationship between lobular organization and transcriptomics, we computed a correlation matrix of the gene expression patterns between the lROIs. The matrix highlights how the clusters are situated along the anterior-to-posterior axis (Fig. 3B). A rooted-leaf dendrogram (as implemented inFig. 2B) generated from the correlation matrix splits the lobules into two clusters at the highest level (Fig. 3C; cluster 1: lobules I-IV, V, VI, Crus I; cluster 2: Crus II, lobules VIIb, VIIIa, and VIIIb, IX and X). After the first split, cluster 2 is further clustered into two sets, one consisting of lobules IX and X, and Crus II, and the other lobules VIIb no, VIIa, and VIIIb. To evaluate the probability of reproducing this particular clustering by chance, a bootstrap approach was implemented (n = 10,000, seesection 2), shuffling the gene expression profile within each lROI on each bootstrap. The results of the bootstrap approach demonstrate that the ability of the top genes to correctly order the lROIs is highly significant (p < .00001). The gene expression patterns in the lROIs were submitted to a principal component analysis. Consistent with the previous results, the first component captured the split at the horizontal fissure in which regions above the horizontal fissure have a positive loading score and regions below have a negative loading score (Fig. 3D). This component accounted for 40% of the overall variance. The second component (not featured at the group level) explained an additional 37%. These results were confirmed at the individual donor level. The split at the horizontal fissure is captured in all six donors in either the first or the second component (Fig. S5). Clustering was also performed at the single-sample level to remove any spatial bias that may emerge as a result of parcellating the data. At the highest level, samples were grouped into 1 of 3 clusters with one cluster highlighting a boundary at the horizontal fissure (shown in green inFig. S6). The genetic organization of the human cerebellum along the anterior-to-posterior axis is consistent with a similar differentiation observed in the animal literature (Fig. 3E). One difference of note is that our genetic analyses reveal a sharp boundary in transcriptomic patterns across the horizontal fissure (Fig. 3F), while the boundary traversing Purkinje cell production (light checkered to heavy checkered patterns) occurs at the primary fissure (*pri*inFig. 3E) in the adult rat cerebellum (Altman & Bayer, 1985).

### 3.3 Filtering for cortex-specific genes captures an additional axis of transcriptomic variation that aligns with functional gradients of the cerebellum

To examine the impact of gene selection methods on the present results, we also applied a ‘cortex-specific’ filter (Burt et al., 2018) to our gene set and repeated the previous analyses. Using samples collected byFagerberg et al. (2014), these ‘cortex-specific’ genes (n = 2,413) are expressed in the HCC, but not in other organs or tissues (Genovese et al., 2016). With regards to the AHBA, this set has been used to improve the sensitivity of genes selected by recent studies looking at the relationship between transcriptomics and the human cerebral cortex (Burt et al., 2018;L. King & Weiner, 2024), but has not yet been employed in an analysis of the human cerebellar cortex. We refer to these genes as ‘cortex-specific’ because it is an open question if filtering for these genes also reveals a transcriptomic contribution to the functional layout of the human cerebellum.

**Fig. 4.**
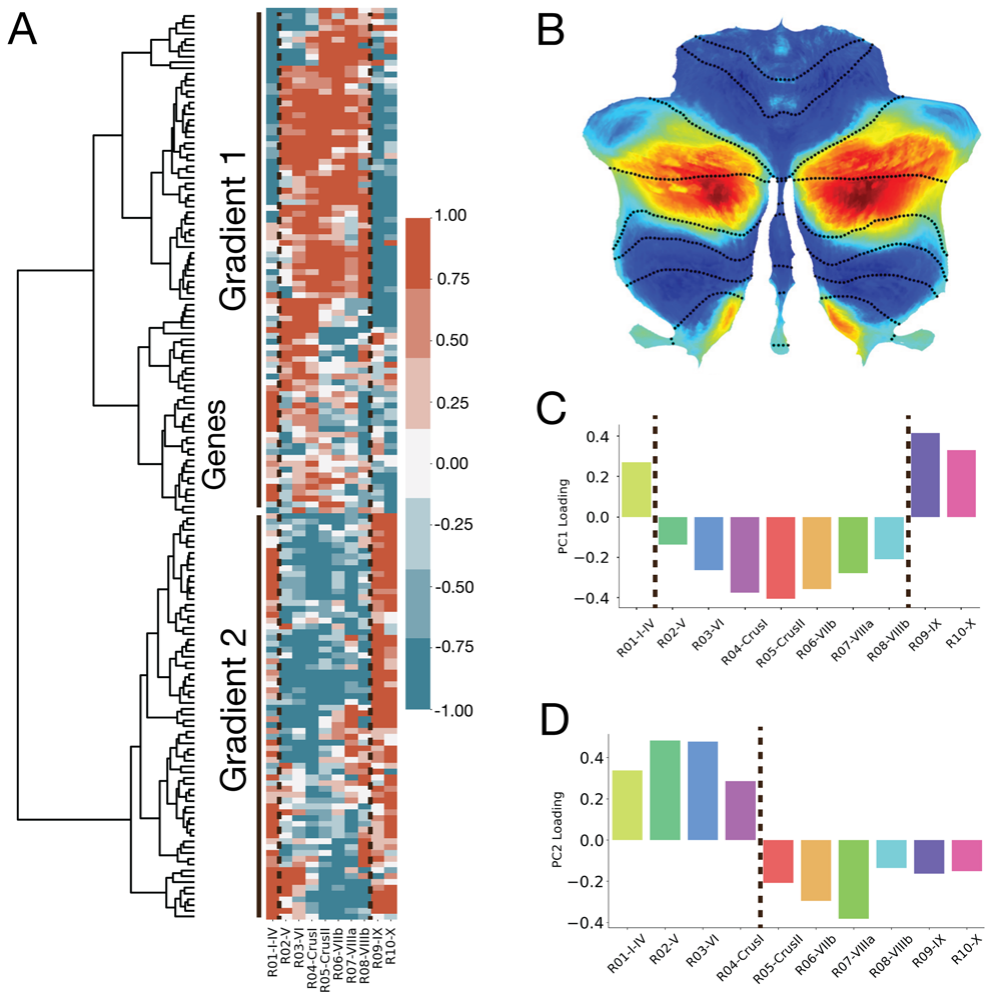
Filtering for cortex-specific genes captures an additional transcriptomic axis that aligns with functional gradients. (A) Filtering for cortex-specific genes,Figure 3Awas re-created where the two dashed lines indicate the boundaries seen in the first principle component shown in (C). (B) Cerebellum functional gradient produced by task activation maps byGuell et al. (2018). Shown here is ‘Gradient 1’ that extends from Default Mode Network and language regions to motor regions in the cerebellum. (C) and (D) PC loading scores as a function of lobule for PC1 (C) and PC2 (D). The first PC captures a crossover at the tail-end regions that are closest in anatomical proximity due to the curvature of the cerebellum. The highest absolute PC loads scores at lobules Crus I and Crus II, while PC2 is similar to that ofFigure 3D, capturing a crossover at the boundary of the horizontal fissure (vertical dashed line).

Filtering for ‘cortex-specific’ genes resulted in a new set of genes (n = 157); only a minority of which (n = 28) overlapped with our original set. Repeating our agglomerative hierarchical clustering approach on this set of ‘cortex-specific’ genes resulted in two primary gradients that exhibit spatial patterns distinct from the three gradients identified previously from the non-filtered gene set (Fig. 4Avs.Fig. 3A, respectively). The first gradient consists of genes highly expressed in lobules more caudal, or furthest, from the brainstem (lobules V-VIIIb, CrusI, and CrusII) and genes lowly expressed in regions rostral, or closest, to the brainstem (lobules I-IV, IX, and X). The second gradient reflects the inverse of this pattern. Based on prior neuroimaging studies of the cerebellum, gradient 1 appears to align with functional gradients of the cerebellum (Guell et al., 2018;Katsumi et al., 2023), such as the primary functional gradient produced byGuell et al. (2018)(shown inFig. 4B), while gradient 2 is inversely related to this pattern.

PCA revealed two anatomical axes captured by this gene set. The first component highlights a gradient from anterior (Fig. 4B: lobules IV, IX-X) versus posterior (Fig. 4C: lobules V-VIII, Crus I, and Crus II) loci relative to the brainstem. This anterior-to-posterior gradient looks remarkably similar across our transcriptomic data and intracerebellar patterns of connectivity obtained using resting-state fMRI (Guell et al., 2018;Fig. 4B). The second component highlights the same trend seen in the first component of the original gene set (Fig. 4D), with a boundary at the horizontal fissure separating anterior versus posterior regions. The first component accounted for 46% of the variance, and the second component accounted for 30% of the variance.

## 4 Discussion

After implementing a data-driven approach to examine a potential role of gene expression to the anatomical and functional organization of the adult human cerebellum, we observed three key findings. First, transcriptomic gradients align with anatomically defined parcellations of the human cerebellum. Second, distinct transcriptomic gradients correspond to an anatomical division of the human cerebellar cortex, with a prominent boundary at the horizontal fissure. Third, alignment with functional gradients of the cerebellum only occurs when genes are filtered to cortex-specific genes. We discuss these findings in the following two sections which further unpack how i) transcriptomic gradients primarily correspond with the lobular organization of the human cerebellum and ii) application of cortically-filtered genes correspond with functional gradients, rather than discrete functional boundaries, of the human cerebellum.

### 4.1 Transcriptomic gradients correspond with the lobular organization of the human cerebellum

Implementing a data-driven approach blind to the lobules and fissures of the cerebellum encapsulated a broad anatomical differentiation of genetic expression along the anterior-posterior axis of the human cerebellum, corresponding to three distinct genetic gradients. Gradient 1 captures a sharp transcriptomic boundary at the horizontal fissure. The other two gradients were mirror reflections of one another, consisting of a set of genes in Gradient 2 that were highly expressed in lobules V-VIIIb and weakly expressed in lobules I-IV, IX, and X, or vice-versa (Gradient 3). Furthermore, gene expression in vermal regions (medial) of the cerebellum exhibited a distinct pattern of genetic expression compared to hemispheric regions (lateral), suggesting differentiation along the medial-to-lateral (ML) axis of the human cerebellum (Figs. 3Fand S7). This finding corroborates what we know from animal models, namely that the hemispheres have a distinct foliation pattern compared to the vermis (Cheng et al., 2010), as well as notable differences in cerebellar cytoarchitecture (Lange, 1982) These results illustrate an alignment between transcriptomic gradients of the human cerebellum and an anatomical organization, at the level of cerebellar lobules.

However, there are a few limitations that are worth noting. First, sample distribution in the AHBA is discontinuous, resulting in irregular coverage across cerebellar ROIs. For example, lobules VI, VIIIa, IX, and X were the only vermal regions that were sampled. As such, we should be cautious about drawing strong conclusions about differentiation along the medial-to-lateral axis (Fig. S7). Second, the registration of gene expression to MNI space may bias alignment to structural MRI data rather than functional MRI data. However, we emphasize that our approach is consistent with widely used computational toolboxes that include the JuGEx (https://ebrains.eu/service/jugex/) toolbox, which aims to examine the relationship between gene expression analyses and human brain atlases. For example,Bludau et al. (2018) align AHBA and functional, as well as cytoarchitectonic, data to the MNI152 space similar to the approach that we adopt in the present work. It is unclear whether incorporating both anatomical and functional data for alignment would significantly impact our current results. While this approach could potentially be beneficial, it would still necessitate analyses in stereotaxic space due to the computational demands of segmenting and reconstructing individual cerebella (Sereno et al., 2020). However, despite these limitations, the present analyses were sensitive enough to identify a prominent transcriptomic gradient along the lateral-medial axis. Additionally, anatomical subdivisions at the horizontal fissure were identifiable in either principal component 1 or 2 of all 6 subjects (Fig. S5). Lastly, while only two donors had bi-hemispheric coverage, analyses across both hemispheres shows that gene expression is highly correlated (Fig. S7). It is important to note that as new datasets become available (Aldinger et al., 2021;Ament et al., 2023), human brain transcriptomic data will better capture the breadth of the cerebellum’s molecular repertoire, allowing for better inferences about genetic differentiation along anatomical and functional gradients of the human cerebellum.

The finding that transcriptomic gradients most closely correspond with the lobular organization of the human cerebellum is consistent with previous approaches. For example, cerebellar architecture has been characterized by patterns of lobes and lobules (Marzban & Hawkes, 2011) in which these patterns are under strong genetic control and are highly reproducible across species (White & Sillitoe, 2013). Embryological studies have highlighted that the cerebellar fissures demarcate different developmental trajectories within the cerebellum (White & Sillitoe, 2013). Specifically, the primary fissure, which separates the anterior and posterior lobes, has been shown to delineate two distinct gene expression profiles (En1 and En2) between lobules I-V and lobules VI-X (White & Sillitoe, 2013). More recently, the correspondence between transcriptomic gradients and lobular organization has been corroborated in the developing cerebellum.Liu et al. (2022)identified a tissue gradient across the anterior-to-posterior axis of the developing cerebellum, which varied as a function of lobular position. These findings provide additional evidence that lobulation in the cerebellum is under genetic control and is functionally relevant (Bailey et al., 2014).

While it is frequently noted that the cerebellar cortex is cytoarchitectonically homogeneous (Ito, 2006;Witter & De Zeeuw, 2015), there is also a recent acknowledgment that there are microarchitectonic differences in the density of Purkinje cells across the cerebellar cortex (Cerminara et al., 2015). These differences are also found along an anterior-posterior axis, in which Purkinje cell density decreases from the anterior to posterior lobes of the cerebellum and the morphology of these cells is determined by their anatomical location (Armstrong & Schild, 1970;Cerminara et al., 2015). Additionally, cell density has been proposed as a microarchitectonic feature linking transcriptomic gradients to macroanatomical and functional gradients across areas of the cerebral cortex (Gomez et al., 2020;L. King & Weiner, 2024), which may be shared across both structures. Thus, the transcriptomic gradients identified here may be related to differences in the density and morphology of Purkinje cells or cellular density across layers along an anterior-posterior axis in the human cerebellum, a hypothesis that can be explored in future research.

### 4.2 Transcriptomic gradients do not correspond with functional parcellations of the human cerebellum

We did not observe a correspondence between transcriptomic gradients and functional parcellations of the human cerebellum. Given that lobular boundaries do not strongly align with functional boundaries in the human cerebellum (M. King et al., 2019), it is perhaps not surprising that transcriptomic gradients do not align with both layouts. Of course, an important caveat to note is that functional boundaries in the human cerebellum are currently defined using BOLD-based activation. This is only one measure of brain activity and it likely does not fully capture the complete spectrum of cerebellar functional organization. We understand from molecular techniques, including aldolase C (zebrin II) expression in Purkinje cells, that anatomical organization in the cerebellum can represent functional units known as “microzones” (Cerminara et al., 2015). These units, which have been primarily identified in rodents (Sugihara & Shinoda, 2004) and primates (Leclerc et al., 1990), form parasagittal zones across the cerebellar cortex, and vary in functionality (Apps & Hawkes, 2009).

“Microzones” have not as yet been identified in the human cerebellar cortex, therefore our current understanding of functional boundaries is dependent on BOLD-based approaches. However, we believe that it is unlikely that these zones align with BOLD-based functional boundaries because the cerebellar hemodynamic signal primarily reflects mossy and climbing fiber inputs (Lauritzen, 2001) with single climbing fibers targeting Purkinje cells within a zone, and more diffuse mossy fiber innervation spanning multiple zonal regions (Hawkes, 1997). Therefore, it is unlikely that the functional boundaries are tightly aligned with this zonal compartmentation. Furthermore, and related to the latter point, mossy and climbing fibers provide input from extra-cerebellar structures (e.g., cerebral cortex, spinal cord, and brain stem) (Thomsen et al., 2004), and we know from resting-state fMRI that there is a strong functional association between distinct regions of the cerebral cortex and corresponding regions of the human cerebellum (Buckner et al., 2011). We also know from task-based fMRI that a sizable proportion (~60%) of the task-evoked variance in the cerebellum can be explained by activity in the cerebral cortex (M. King et al., 2023). Therefore, it is possible that functional boundaries in the human cerebellum are attributable to cerebro-cerebellar connections and that these may not be under strong genetic control, but rather are likely shaped postnatally through experience-dependent activity.

### 4.3 Transcriptomic and functional gradients are aligned for cortex-specific genes

Interestingly, when we applied ‘cortex-specific’ filtering for gene selection, we observed an alignment between transcriptomic and functional gradients of the cerebellum. One possible explanation for the correspondence between transcriptomic and functional gradients is the fact that the ‘cortex-specific’ genes are common across both structures with shared biological processes such as synaptic maintenance and function, as suggested by previous enrichment analyses (Burt et al., 2018;L. King & Weiner, 2024). Furthermore, while BOLD-based measures of function do not directly confer with anatomical divisions in the cerebellum, relation to functional gradients with this gene set may, in part, be due to the anatomical properties that underlie function. With recent studies examining the spatial patterns of transcriptomic gradients of the HCC (Dear et al., 2024;L. King & Weiner, 2024;Vogel et al., 2024;Wagstyl et al., 2024), it will be interesting to see how cerebellar gradients overlap with cortical gradients. Results from the ‘cortex-specific’ filtered set in the cerebellum suggest that there may be a correspondence to gradients in the cortex, which has been found to be continuous in prior work (Vogel et al., 2024).

## 5 Conclusion

The present findings demonstrate that genetic expression gradients align with the anterior-posterior layout of the adult human cerebellum, a result consistent with observations from previous studies involving other mammalian species (White & Sillitoe, 2013). However, using cortically-defined genes, we find a strong relationship between transcriptomic gradients and smooth BOLD-based functional gradients (Guell et al., 2018;Katsumi et al., 2023), rather than discrete parcellated boundaries. Two explanations may account for this dissociation: 1) Unlike the cerebral cortex, anatomical and functional organization are not entirely aligned in the human cerebellum, and thus, genetic expression patterns are unlikely to be simultaneously aligned with both, and 2) Functional organization in the human cerebellum, as currently defined with fMRI, is strongly defined by cerebro-cerebellar connections (M. King et al., 2023) that are likely not under genetic control but rather shaped postnatally through experience-dependent functions (Haldipur et al., 2019).

## Supplementary Material

Supplementary Material

## Data Availability

Data are available to download athttps://human.brain-map.org/static/download. Code is available athttps://github.com/maedbhk/cerebellum_transcriptomics. Notebooks are available to preprocess the data and to visualize the figures. Further information and other inquiries should be directed to and will be fulfilled by Maedbh King (maedbhking@gmail.com) or Leana King (leana.king@berkeley.edu).

## References

[b1] Aldinger , K. A. , Thomson , Z. , Phelps , I. G. , Haldipur , P. , Deng , M. , Timms , A. E. , Hirano , M. , Santpere , G. , Roco , C. , Rosenberg , A. B. , Lorente-Galdos , B. , Gulden , F. O. , O’Day , D. , Overman , L. M. , Lisgo , S. N. , Alexandre , P. , Sestan , N. , Doherty , D. , Dobyns , W. B. , … Millen , K. J. ( 2021 ). Spatial and cell type transcriptional landscape of human cerebellar development . Nature Neuroscience , 24 ( 8 ), 1163 – 1175 . 10.1038/s41593-021-00872-y 34140698 PMC8338761

[b2] Altman , J. , & Bayer , S. A. ( 1985 ). Embryonic development of the rat cerebellum. III. Regional differences in the time of origin, migration, and settling of Purkinje cells . The Journal of Comparative Neurology , 231 ( 1 ), 42 – 65 . 10.1002/cne.902310105 3968228

[b3] Ament , S. A. , Cortes-Gutierrez , M. , Herb , B. R. , Mocci , E. , Colantuoni , C. , & McCarthy , M. M. ( 2023 ). A single-cell genomic atlas for maturation of the human cerebellum during early childhood . Science Translational Medicine , 15 ( 721 ), eade1283 . 10.1126/scitranslmed.ade1283 37824600

[b4] Apps , R. , & Hawkes , R. ( 2009 ). Cerebellar cortical organization: A one-map hypothesis . Nature Reviews. Neuroscience , 10 ( 9 ), 670 – 681 . 10.1038/nrn2698 19693030

[b5] Armstrong , D. M. , & Schild , R. F. ( 1970 ). A quantitative study of the Purkinje cells in the cerebellum of the albino rat . The Journal of Comparative Neurology , 139 ( 4 ), 449 – 456 . 10.1002/cne.901390405 4917615

[b6] Arnatkevičiūtė , A. , Fulcher , B. D. , & Bellgrove , M. A. ( 2021 ). Imaging transcriptomics of brain disorders . Biological Psychiatry Global Open Science , 2 ( 4 ), 319 - 331 . https://www.sciencedirect.com/science/article/pii/S2667174321001191 36324650 10.1016/j.bpsgos.2021.10.002 PMC9616271

[b7] Arnatkevičiūtė , A. , Fulcher , B. D. , & Fornito , A. ( 2019 ). A practical guide to linking brain-wide gene expression and neuroimaging data . NeuroImage , 189 , 353 – 367 . 10.1016/j.neuroimage.2019.01.011 30648605

[b8] Arnatkevičiūtė , A. , Fulcher , B. D. , Oldham , S. , Tiego , J. , Paquola , C. , Gerring , Z. , Aquino , K. , Hawi , Z. , Johnson , B. , Ball , G. , Klein , M. , Deco , G. , Franke , B. , Bellgrove , M. A. , & Fornito , A. ( 2021 ). Genetic influences on hub connectivity of the human connectome . Nature Communications , 12 ( 1 ), 4237 . 10.1038/s41467-021-24306-2 PMC8271018 34244483

[b9] Arnatkevičiūtė , A. , Markello , R. D. , Fulcher , B. D. , Misic , B. , & Fornito , A. ( 2023 ). Toward best practices for imaging transcriptomics of the human brain . Biological Psychiatry , 93 ( 5 ), 391 – 404 . 10.1016/j.biopsych.2022.10.016 36725139

[b10] Ashida , R. , Cerminara , N. L. , Brooks , J. , & Apps , R. ( 2018 ). Principles of organization of the human cerebellum: Macro- and microanatomy . Handbook of Clinical Neurology , 154 , 45 – 58 . 10.1016/B978-0-444-63956-1.00003-5 29903451

[b11] Bailey , K. , Rahimi Balaei , M., Mannan , A. , Del Bigio , M. R. , & Marzban , H. ( 2014 ). Purkinje cell compartmentation in the cerebellum of the lysosomal acid phosphatase 2 mutant mouse (nax - naked-ataxia mutant mouse) . PLoS One , 9 ( 4 ), e94327 . 10.1371/journal.pone.0094327 24722417 PMC3983142

[b200] Bludau , S. , Mühleisen , T. W. , Eickhoff , S. B. , Hawrylycz , M. J. , Cichon , S. , & Amunts , K . ( 2018 ). Integration of transcriptomic and cytoarchitectonic data implicates a role for MAOA and TAC1 in the limbic-cortical network . Brain Structure and Function , 223 ( 5 ), 2335 – 2342 . 10.1007/s00429-018-1620-6 29478144 PMC5968065

[b12] Buckner , R. L. , Krienen , F. M. , Castellanos , A. , Diaz , J. C. , & Yeo , B. T. T. ( 2011 ). The organization of the human cerebellum estimated by intrinsic functional connectivity . Journal of Neurophysiology , 106 ( 5 ), 2322 – 2345 . 10.1152/jn.00339.2011 21795627 PMC3214121

[b13] Burt , J. B. , Demirtaş , M. , Eckner , W. J. , Navejar , N. M. , Ji , J. L. , Martin , W. J. , Bernacchia , A. , Anticevic , A. , & Murray , J. D. ( 2018 ). Hierarchy of transcriptomic specialization across human cortex captured by structural neuroimaging topography . Nature Neuroscience , 21 ( 9 ), 1251 – 1259 . 10.1038/s41593-018-0195-0 30082915 PMC6119093

[b14] Cerminara , N. L. , Lang , E. J. , Sillitoe , R. V. , & Apps , R. ( 2015 ). Redefining the cerebellar cortex as an assembly of non-uniform Purkinje cell microcircuits . Nature Reviews. Neuroscience , 16 ( 2 ), 79 – 93 . 10.1038/nrn3886 25601779 PMC4476393

[b15] Cheng , Y. , Sudarov , A. , Szulc , K. U. , Sgaier , S. K. , Stephen , D. , Turnbull , D. H. , & Joyner , A. L. ( 2010 ). The Engrailed homeobox genes determine the different foliation patterns in the vermis and hemispheres of the mammalian cerebellum . Development (Cambridge, England) , 137 ( 3 ), 519 – 529 . 10.1242/dev.027045 20081196 PMC2858911

[b16] Dear , R. , Wagstyl , K. , Seidlitz , J. , Markello , R. D. , Arnatkevičiūtė , A. , Anderson , K. M. , Bethlehem , R. A. I. , Raznahan , A. , Bullmore , E. T. , Vértes , P. E. , & Lifespan Brain Chart Consortium . ( 2024 ). Cortical gene expression architecture links healthy neurodevelopment to the imaging, transcriptomics and genetics of autism and schizophrenia . Nature Neuroscience , 27 ( 6 ), 1075 – 1086 . 10.1038/s41593-024-01624-4 38649755 PMC11156586

[b17] Deco , G. , Kringelbach , M. L. , Arnatkevičiūtė , A. , Oldham , S. , Sabaroedin , K. , Rogasch , N. C. , Aquino , K. M. , & Fornito , A. ( 2021 ). Dynamical consequences of regional heterogeneity in the brain’s transcriptional landscape . Science Advances , 7 ( 29 ), eabf4752 . 10.1126/sciadv.abf4752 34261652 PMC8279501

[b18] Diedrichsen , J. ( 2006 ). A spatially unbiased atlas template of the human cerebellum . NeuroImage , 33 ( 1 ), 127 – 138 . 10.1016/j.neuroimage.2006.05.056 16904911

[b19] Diedrichsen , J. , Balsters , J. H. , Flavell , J. , Cussans , E. , & Ramnani , N. ( 2009 ). A probabilistic MR atlas of the human cerebellum . NeuroImage , 46 ( 1 ), 39 – 46 . 10.1016/j.neuroimage.2009.01.045 19457380

[b20] Diedrichsen , J. , & Zotow , E. ( 2015 ). Surface-based display of volume-averaged cerebellar imaging data . PLoS One , 10 ( 7 ), e0133402 . 10.1371/journal.pone.0133402 26230510 PMC4521932

[b21] Fagerberg , L. , Hallström , B. M. , Oksvold , P. , Kampf , C. , Djureinovic , D. , Odeberg , J. , Habuka , M. , Tahmasebpoor , S. , Danielsson , A. , Edlund , K. , Asplund , A. , Sjöstedt , E. , Lundberg , E. , Szigyarto , C. A.-K. , Skogs , M. , Takanen , J. O. , Berling , H. , Tegel , H. , Mulder , J. , … Uhlén , M. ( 2014 ). Analysis of the human tissue-specific expression by genome-wide integration of transcriptomics and antibody-based proteomics . Molecular & Cellular Proteomics: MCP , 13 ( 2 ), 397 – 406 . 10.1074/mcp.M113.035600 24309898 PMC3916642

[b22] Genovese , G. , Fromer , M. , Stahl , E. A. , Ruderfer , D. M. , Chambert , K. , Landén , M. , Moran , J. L. , Purcell , S. M. , Sklar , P. , Sullivan , P. F. , Hultman , C. M. , & McCarroll , S. A. ( 2016 ). Increased burden of ultra-rare protein-altering variants among 4,877 individuals with schizophrenia . Nature Neuroscience , 19 ( 11 ), 1433 – 1441 . 10.1038/nn.4402 27694994 PMC5104192

[b23] Gomez , J. , Zhen , Z. , & Weiner , K. ( 2018 ). Human visual cortex is organized along two genetically opposed hierarchical gradients with unique developmental and evolutionary origins . bioRxiv , 200 , 495143 . 10.1101/495143 PMC6634416 31269028

[b24] Gomez , J. , Zhen , Z. , & Weiner , K. S. ( 2019 ). Human visual cortex is organized along two genetically opposed hierarchical gradients with unique developmental and evolutionary origins . PLoS Biology , 17 ( 7 ), e3000362 . 10.1371/journal.pbio.3000362 31269028 PMC6634416

[b25] Gomez , J. , Zhen , Z. , & Weiner , K. S. ( 2020 ). Opposed transcriptomic gradients contribute to both the arealization of human visual cortex and the topological layout of its orthogonal maps . Journal of Vision , 20 ( 11 ), 343 . 10.1167/jov.20.11.343

[b26] Gomez , J. , Zhen , Z. , & Weiner , K. S. ( 2021 ). The relationship between transcription and eccentricity in human V1 . Brain Structure & Function , 226 ( 9 ), 2807 – 2818 . 10.1007/s00429-021-02387-5 34618233

[b27] Guell , X. , Schmahmann , J. D. , Gabrieli , J. D. E. , & Ghosh , S. S. ( 2018 ). Functional gradients of the cerebellum . eLife , 7 , e36652 . 10.7554/eLife.36652 30106371 PMC6092123

[b29] Haldipur , P. , Aldinger , K. A. , Bernardo , S. , Deng , M. , Timms , A. E. , Overman , L. M. , Winter , C. , Lisgo , S. N. , Razavi , F. , Silvestri , E. , Manganaro , L. , Adle-Biassette , H. , Guimiot , F. , Russo , R. , Kidron , D. , Hof , P. R. , Gerrelli , D. , Lindsay , S. J. , Dobyns , W. B. , … Millen , K. J. ( 2019 ). Spatiotemporal expansion of primary progenitor zones in the developing human cerebellum . Science , 366 ( 6464 ), 454 – 460 . 10.1126/science.aax7526 31624095 PMC6897295

[b30] Haldipur , P. , Millen , K. J. , & Aldinger , K. A. ( 2022 ). Human cerebellar development and transcriptomics: Implications for neurodevelopmental disorders . Annual Review of Neuroscience , 45 , 515 – 531 . 10.1146/annurev-neuro-111020-091953 PMC9271632 35440142

[b31] Hawkes , R. ( 1997 ). An anatomical model of cerebellar modules . Progress in Brain Research , 114 , 39 – 52 . https://www.ncbi.nlm.nih.gov/pubmed/9193137 9193137 10.1016/s0079-6123(08)63357-9

[b32] Hawrylycz , M. J. , Lein , E. S. , Guillozet-Bongaarts , A. L. , Shen , E. H. , Ng , L. , Miller , J. A. , van de Lagemaat , L. N. , Smith , K. A. , Ebbert , A. , Riley , Z. L. , Abajian , C. , Beckmann , C. F. , Bernard , A. , Bertagnolli , D. , Boe , A. F. , Cartagena , P. M. , Chakravarty , M. M. , Chapin , M. , Chong , J. , … Jones , A. R. ( 2012 ). An anatomically comprehensive atlas of the adult human brain transcriptome . Nature , 489 ( 7416 ), 391 – 399 . 10.1038/nature11405 22996553 PMC4243026

[b33] Ito , M. ( 2006 ). Cerebellar circuitry as a neuronal machine . Progress in Neurobiology , 78 ( 3-5 ), 272 – 303 . 10.1016/j.pneurobio.2006.02.006 16759785

[b34] Katsumi , Y. , Zhang , J. , Chen , D. , Kamona , N. , Bunce , J. G. , Hutchinson , J. B. , Yarossi , M. , Tunik , E. , Dickerson , B. C. , Quigley , K. S. , & Barrett , L. F. ( 2023 ). Correspondence of functional connectivity gradients across human isocortex, cerebellum, and hippocampus . Communications Biology , 6 ( 1 ), 401 . 10.1038/s42003-023-04796-0 37046050 PMC10097701

[b35] King , L. , & Weiner , K. S. ( 2024 ). Transcriptomic contributions to a modern cytoarchitectonic parcellation of the human cerebral cortex . Brain Structure & Function , 229 ( 4 ), 919 – 936 . 10.1007/s00429-023-02754-4 38492042

[b36] King , M. , Hernandez-Castillo , C. R. , Poldrack , R. A. , Ivry , R. B. , & Diedrichsen , J. ( 2019 ). Functional boundaries in the human cerebellum revealed by a multi-domain task battery . Nature Neuroscience , 22 ( 8 ), 1371 – 1378 . 10.1038/s41593-019-0436-x 31285616 PMC8312478

[b201] King , M. , Shahshahani , L. , Ivry , R. B. , & Diedrichsen , J. ( 2023 ). A task-general connectivity model reveals variation in convergence of cortical inputs to functional regions of the cerebellum . eLife , 12 , e81511 . 10.7554/eLife.81511 37083692 PMC10129326

[b37] Lange , W. ( 1982 ). An anatomical study summarizing the differences in Purkinje cell, granule cell and Golgi cell density and cell size between the vermis and hemispheres of the cerebellum . In S. L. P. Chan-Palay (Ed.), The cerebellum: New vistas (pp. 93 – 105 ). Springer International Publishing . https://link.springer.com/book/9783642685620

[b38] Larsell , O. ( 1947 ). The development of the cerebellum in man in relation to its comparative anatomy . The Journal of Comparative Neurology , 87 , 85 – 129 . 10.1002/cne.900870203 20267600

[b39] Lauritzen , M. ( 2001 ). Relationship of spikes, synaptic activity, and local changes of cerebral blood flow . Journal of Cerebral Blood Flow and Metabolism: Official Journal of the International Society of Cerebral Blood Flow and Metabolism , 21 ( 12 ), 1367 – 1383 . 10.1097/00004647-200112000-00001 11740198

[b40] Leclerc , N. , Doré , L. , Parent , A. , & Hawkes , R. ( 1990 ). The compartmentalization of the monkey and rat cerebellar cortex: Zebrin I and cytochrome oxidase . Brain Research , 506 ( 1 ), 70 – 78 . 10.1016/0006-8993(90)91200-Z 2154279

[b41] Liu , X. , d’Oleire Uquillas , F., Viaene , A. N. , Zhen , Z. , & Gomez , J. ( 2022 ). A multifaceted gradient in human cerebellum of structural and functional development . Nature Neuroscience , 25 ( 9 ), 1129 – 1133 . 10.1038/s41593-022-01136-z 35982153

[b42] Markello , R. D. , Arnatkevičiūtė , A. , Poline , J.-B. , Fulcher , B. D. , Fornito , A. , & Misic , B. ( 2021 ). Standardizing workflows in imaging transcriptomics with the abagen toolbox . eLife , 10 , e72129 . 10.7554/eLife.72129 34783653 PMC8660024

[b43] Marzban , H. , & Hawkes , R. ( 2011 ). On the architecture of the posterior zone of the cerebellum . Cerebellum , 10 ( 3 ), 422 – 434 . 10.1007/s12311-010-0208-3 20838950

[b44] Miller , J. A. , Ding , S. L. , Sunkin , S. M. , Smith , K. A. , Ng , L. , Szafer , A. , Ebbert , A. , Riley , Z. L. , Royall , J. J. , Aiona , K. , Arnold , J. M. , Bennet , C. , Bertagnolli , D. , Brouner , K. , Butler , S. , Caldejon , S. , Carey , A. , Cuhaciyan , C. , Dalley , R. A. , … Lein , E. S . ( 2014 ). Transcriptional landscape of the prenatal human brain . Nature , 508 ( 7495 ), 199 – 206 . 10.1038/nature13185 24695229 PMC4105188

[b202] Sereno , M. I. , Diedrichsen , J. , Tachrount , M. , Testa-Silva , G. , d’Arceuil , H. , & De Zeeuw , C. ( 2020 ). The human cerebellum has almost 80% of the surface area of the neocortex . Proceedings of the National Academy of Sciences , 117 ( 32 ), 19538 – 19543 . 10.1073/pnas.2002896117 PMC7431020 32723827

[b45] Shine , J. M. , Arnatkevičiūtė , A. , Fornito , A. , & Fulcher , B. D. ( 2022 ). Navigating a complex landscape: Using transcriptomics to parcellate the human cortex . Biological Psychiatry. Cognitive Neuroscience and Neuroimaging , 7 ( 1 ), 3 – 4 . 10.1016/j.bpsc.2021.10.002 34998482

[b46] Sjöstedt , E. , Zhong , W. , Fagerberg , L. , Karlsson , M. , Mitsios , N. , Adori , C. , Oksvold , P. , Edfors , F. , Limiszewska , A. , Hikmet , F. , Huang , J. , Du , Y. , Lin , L. , Dong , Z. , Yang , L. , Liu , X. , Jiang , H. , Xu , X. , Wang , J. , … Mulder , J. ( 2020 ). An atlas of the protein-coding genes in the human, pig, and mouse brain . Science , 367 ( 6482 ), eaay5947 . 10.1126/science.aay5947 32139519

[b47] Stoodley , C. J. , Valera , E. M. , & Schmahmann , J. D. ( 2012 ). Functional topography of the cerebellum for motor and cognitive tasks: An fMRI study . NeuroImage , 59 ( 2 ), 1560 – 1570 . 10.1016/j.neuroimage.2011.08.065 21907811 PMC3230671

[b48] Sugihara , I. , & Shinoda , Y. ( 2004 ). Molecular, topographic, and functional organization of the cerebellar cortex: A study with combined aldolase C and olivocerebellar labeling . Journal of Neuroscience , 24 ( 40 ), 8771 – 8785 . 10.1523/JNEUROSCI.1961-04.2004 15470143 PMC6729951

[b49] Thomsen , K. , Offenhauser , N. , & Lauritzen , M. ( 2004 ). Principal neuron spiking: Neither necessary nor sufficient for cerebral blood flow in rat cerebellum . The Journal of Physiology , 560 ( Pt 1 ), 181 – 189 . 10.1113/jphysiol.2004.068072 15272036 PMC1665203

[b50] Vogel , J. W. , Alexander-Bloch , A. F. , Wagstyl , K. , Bertolero , M. A. , Markello , R. D. , Pines , A. , Sydnor , V. J. , Diaz-Papkovich , A. , Hansen , J. Y. , Evans , A. C. , Bernhardt , B. , Misic , B. , Satterthwaite , T. D. , & Seidlitz , J. ( 2024 ). Deciphering the functional specialization of whole-brain spatiomolecular gradients in the adult brain . Proceedings of the National Academy of Sciences of the United States of America , 121 ( 25 ), e2219137121 . 10.1073/pnas.2219137121 38861593 PMC11194492

[b51] Wagstyl , K. , Adler , S. , Seidlitz , J. , Vandekar , S. , Mallard , T. T. , Dear , R. , DeCasien , A. R. , Satterthwaite , T. D. , Liu , S. , Vértes , P. E. , Shinohara , R. T. , Alexander-Bloch , A. , Geschwind , D. H. , & Raznahan , A. ( 2024 ). Transcriptional cartography integrates multiscale biology of the human cortex . Elife , 12 , RP86933 . 10.7554/elife.86933.2 38324465 PMC10945526

[b52] White , J. J. , & Sillitoe , R. V. ( 2013 ). Development of the cerebellum: From gene expression patterns to circuit maps . Wiley Interdisciplinary Reviews. Developmental Biology , 2 ( 1 ), 149 – 164 . 10.1002/wdev.65 23799634

[b53] Witter , L. , & De Zeeuw , C. I. ( 2015 ). Regional functionality of the cerebellum . Current Opinion in Neurobiology , 33 , 150 – 155 . 10.1016/j.conb.2015.03.017 25884963

[b54] Yeo , B. T. T. , Krienen , F. M. , Sepulcre , J. , Sabuncu , M. R. , Lashkari , D. , Hollinshead , M. , Roffman , J. L. , Smoller , J. W. , Zöllei , L. , Polimeni , J. R. , Fischl , B. , Liu , H. , & Buckner , R. L. ( 2011 ). The organization of the human cerebral cortex estimated by intrinsic functional connectivity . Journal of Neurophysiology , 106 ( 3 ), 1125 – 1165 . 10.1152/jn.00338.2011 21653723 PMC3174820

