# Supplementary material for: Transcriptomic gradients of the human cerebellum
Source: Imaging Neurosci (Camb). 2025 Feb 26;3:imag_a_00494. doi: 10.1162/imag_a_00494 (PMC12319844; doi:10.1162/imag_a_00494)
Supplement: Supplementary Material [file imag_a_00494-supp.pdf]

Supplemental Material

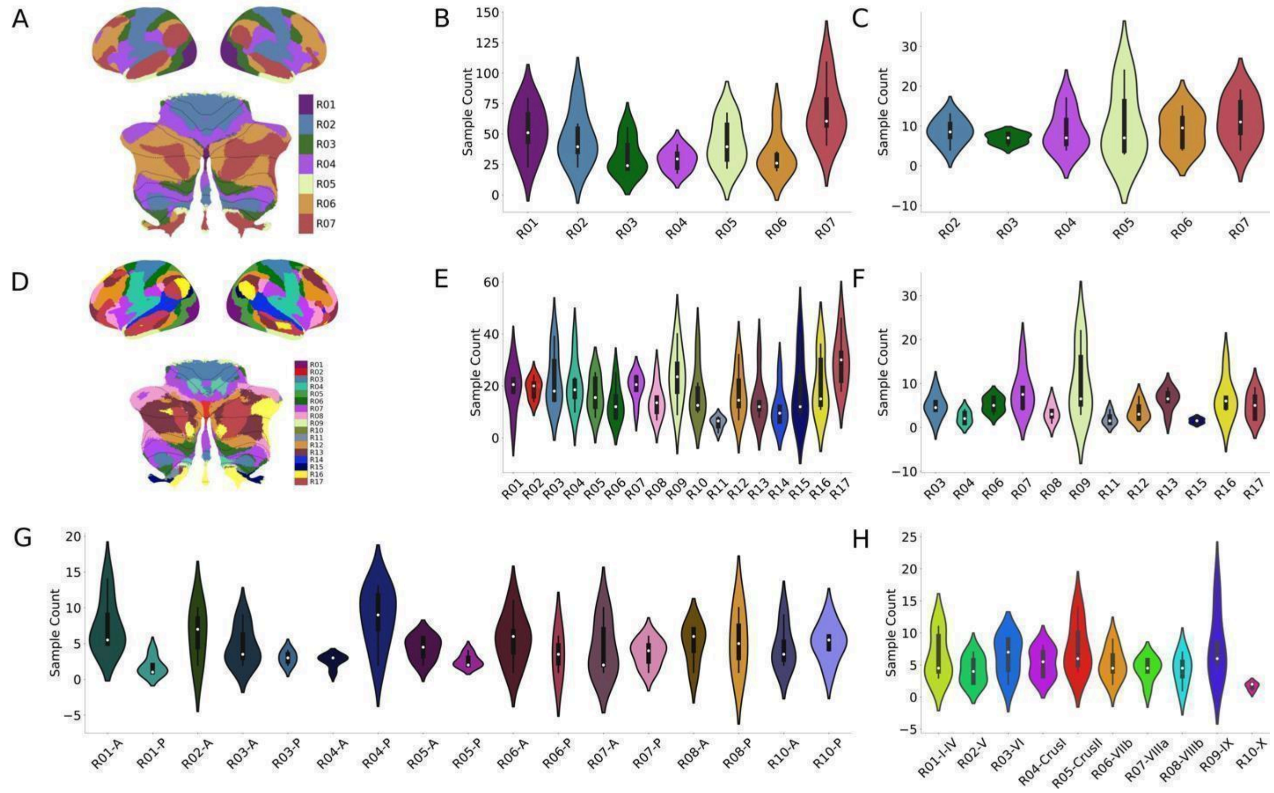

Figure S1. Averaged tissue sample count across regions-of-interest. A) 7-network resting state parcellation of the cerebral cortex and the cerebellum. B) Tissue sample count for the 7 cortical networks and C) 7 cerebellar networks. D) 17-network resting state parcellation of the cerebral cortex and the cerebellum. E) Tissue sample count for the 17 cortical networks and F) 17 cerebellar networks. G) Tissue sample count for the modified task-based atlas of the cerebellum. 3 fROIs did not have any samples, which resulted in a total of 17 fROIs. H) Tissue sample count for the lobular-based anatomical atlas of the cerebellum.

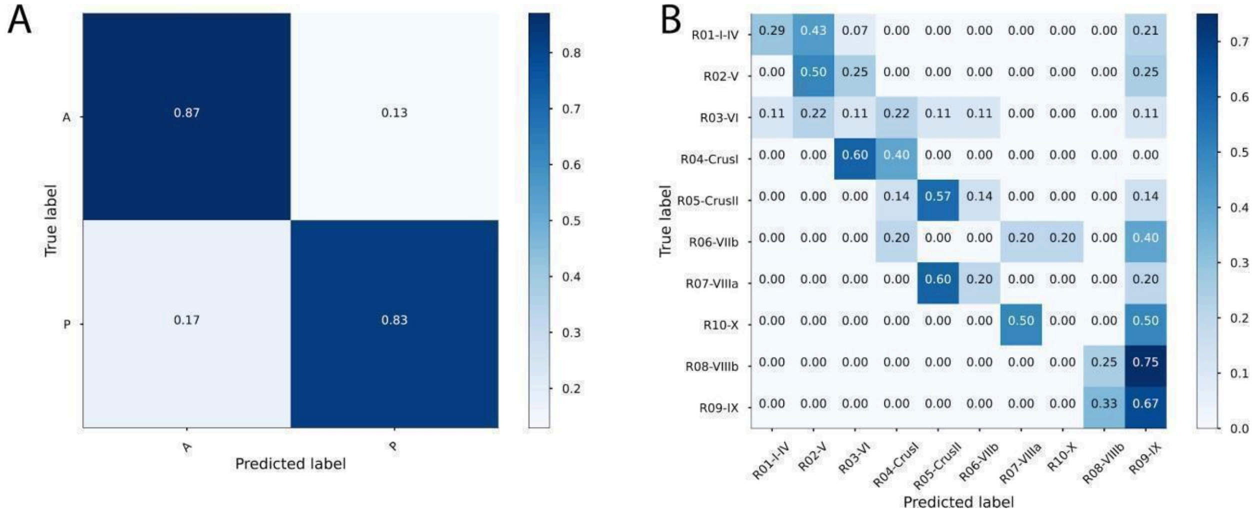

Figure S2. Confusion matrix displaying prediction accuracy for classification of samples across functional (A) and lobular (B) regions. The location of samples across all donors were classified using a cross-validated logistic regression model (Methods). Accuracies are normalized to 1.

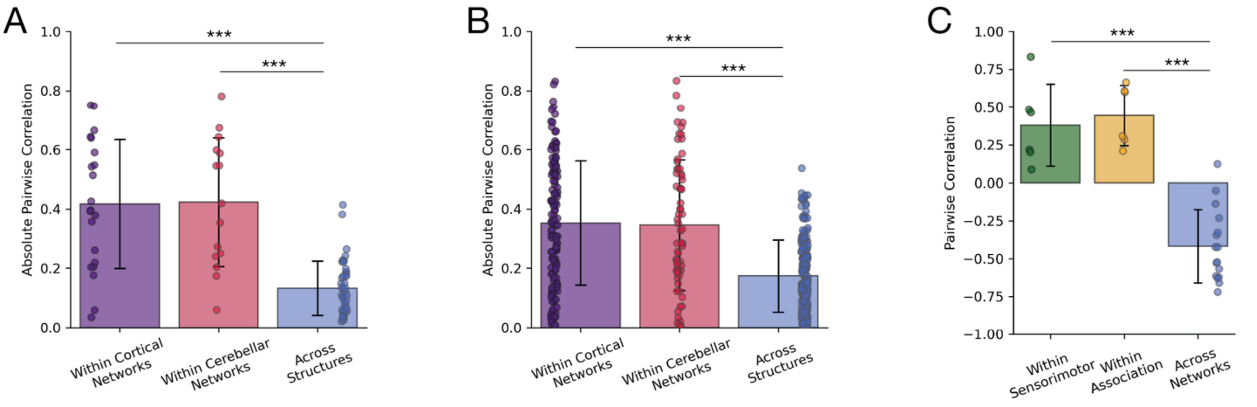

Figure S3. Average pairwise correlations between networks. (A) Using absolute values from the pairwise correlation matrix in Fig 2D, average correlation values within cortical networks (Yeo networks 1-7, purple, mean Pearson's r=0.42) and cerebellar networks (Buckner networks 1-7, red, mean Pearson's r=0.42) were compared to pairwise correlations between cortical and cerebellar networks (blue, mean Pearson's r=0.13) (One-way ANOVA, *** $p < 0.001$). (B) is a replication of (A) but using the Yeo 17-network (purple, mean Pearson's r=0.35) and Buckner 17-network parcellations (red, mean Pearson's r=0.34) to compare within vs. across structure correlations (blue, mean Pearson's r=0.17) shown in Fig 2E. In both (A) and (B), across structure correlations are significantly lower than within structures. (C). Similar to (A) and (B), pairwise correlations are compared between association (Yeo networks 1-4, green, mean Pearson's r=0.38) and sensorimotor networks (Yeo networks 7-10, yellow, mean Pearson's r=0.44) from the Yeo 17 network parcellation and compared to across network correlations (blue, mean Pearson's r=-0.42). Absolute value of pairwise correlations was not taken to highlight the inverse relationship across networks.

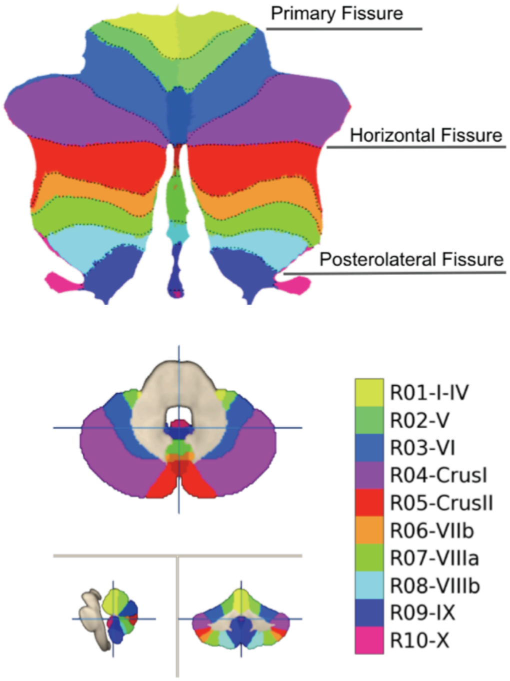

Figure S4. Ten anatomical regions from the spatially unbiased infratentorial atlas[8,9] (SUIT) depicted on a flatmap representation (top) and volume (bottom) of the cerebellum.

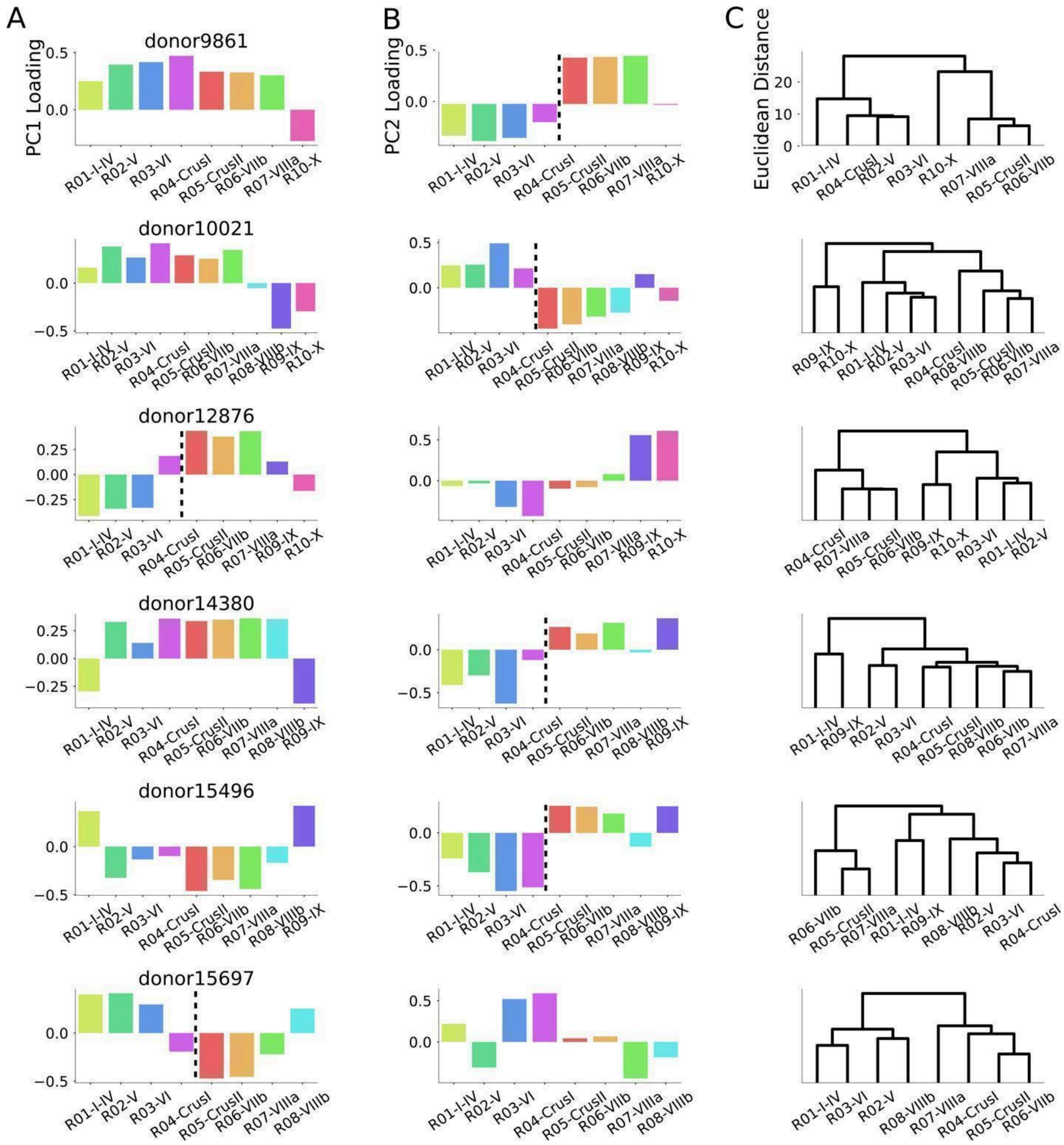

Figure S5. Individual principal component and dendrogram results. A) IROI loadings for the first principal component. B) IROI loadings for the second principal component. 2/6 donors demonstrate the crossover at the horizontal fissure in the first component, while the other 4 demonstrate the crossover in the second component. C) Dendrograms for each individual subject. The dashed line indicates the crossover along the anterior-posterior axis at the horizontal fissure.

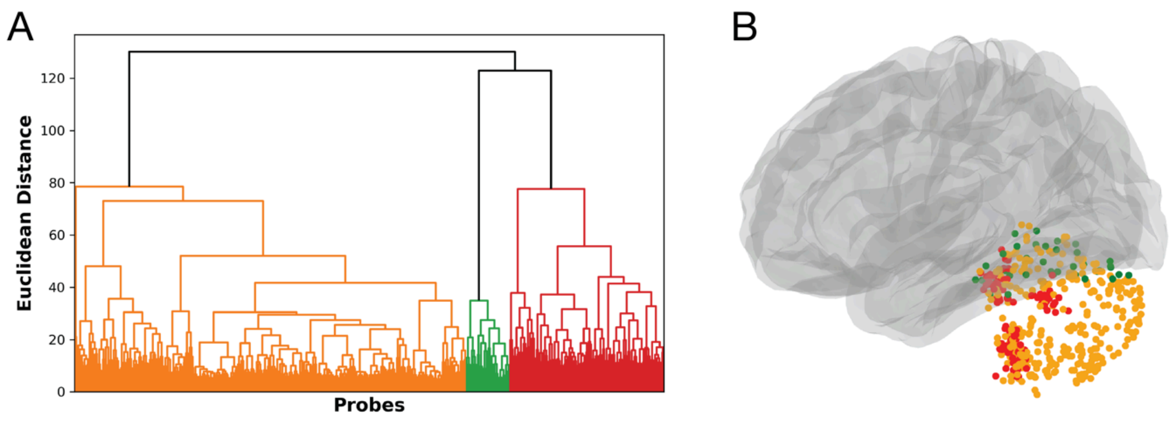

Figure S6. Clustering of cerebellar samples. A) Hierarchical clustering (see *Methods details* section 2.3.1 for more details) of cerebellar samples using the non-filtered set of genes (N=157). Samples were categorized into one of 3 clusters at the highest level (orange, green, and red). B) A 3-D representation of AHBA samples in the cerebellum shown laterally. Samples are color-coded based on their cluster label from the dendrogram in A).

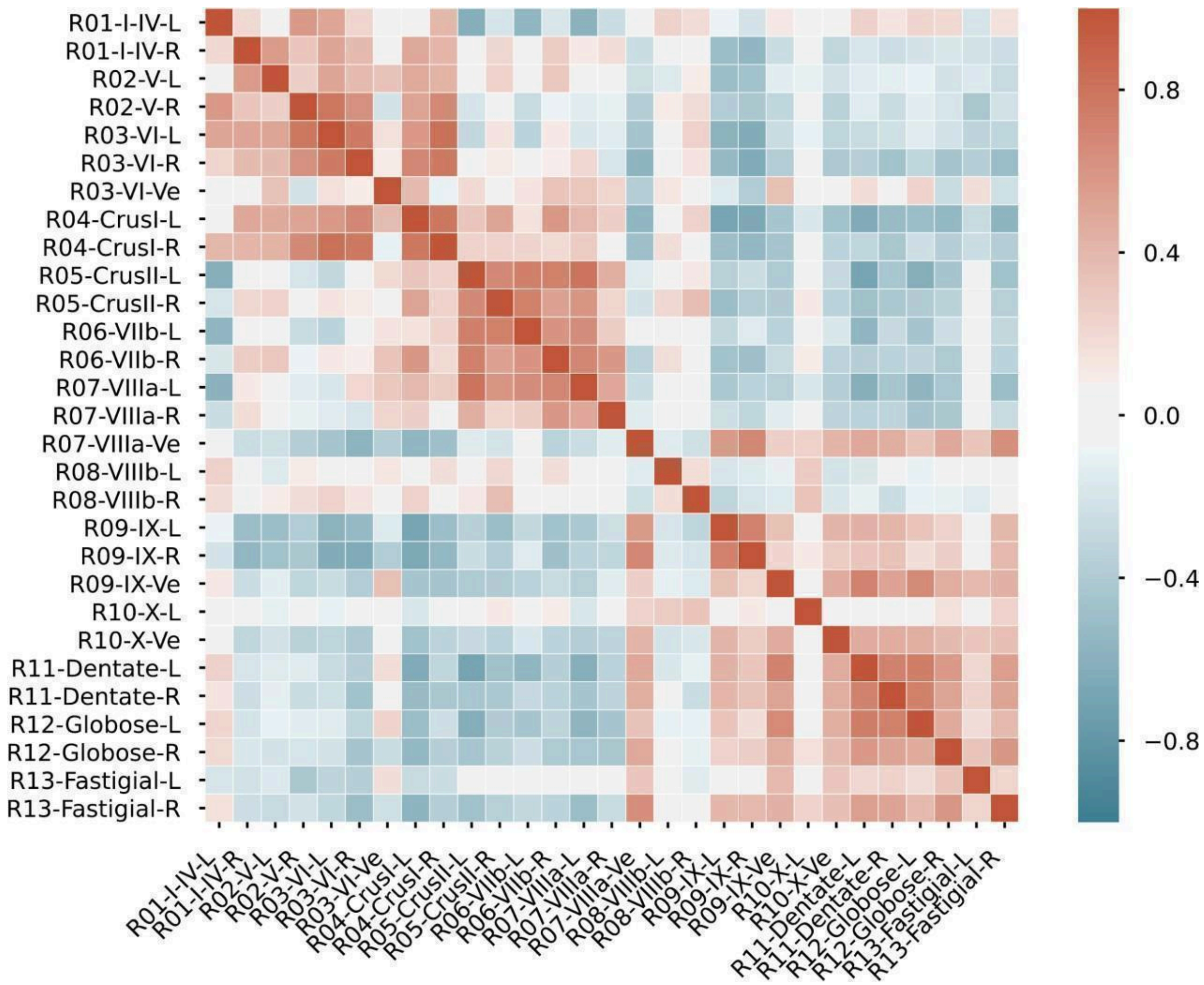

Figure S7. Correlation matrix of gene expression patterns for lobular ROIs (lROIs) in the human cerebellum (SUIT atlas). An anatomical atlas with 34 lROIs was used to determine whether there were genetic gradients along the medial-to-lateral axis of the cerebellum. Lobules were divided into left and right hemispheres. A subset of those regions, VI-X, were also subdivided to include a vermal subregion (see Fig 1A). The correlation matrix also includes deep cerebellar nuclei, which are lROIs that are embedded within the white matter of the cerebellum. Left and right hemispheres of each lROI are highly correlated together. The only vermal regions that were sampled are lobules VI, VIIIa, IX, and X. Despite this small sample size, it appears that at least for lobules VI, VIIIa and X, gene expression in the vermis is very distinct from the left and right hemisphere, perhaps indicating the presence of genetic gradients along the medial-to-lateral axis of the cerebellum. Distance-dependent correlations were removed by regressing out the Euclidean distance between lROIs.

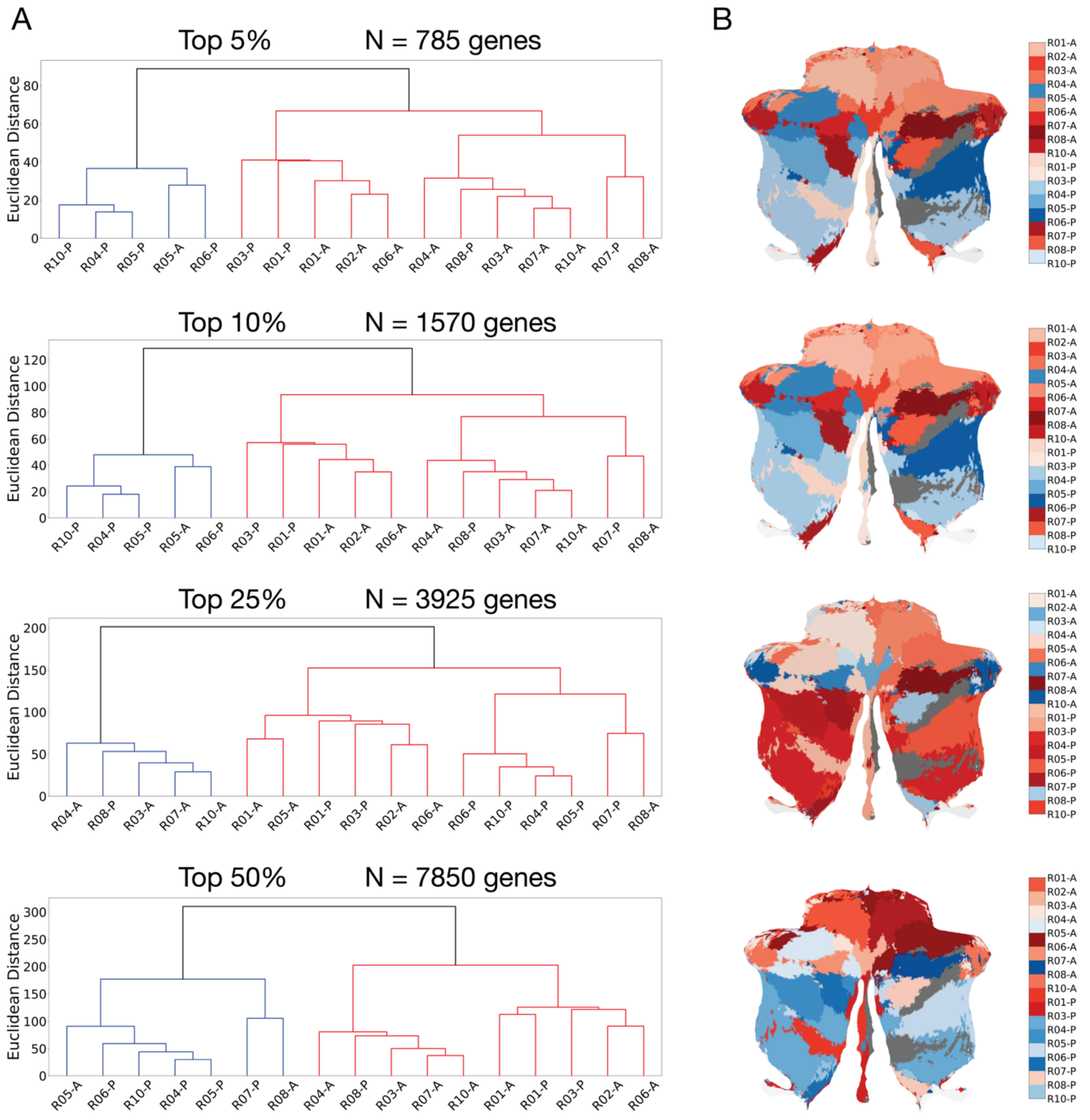

Figure S8. Sensitivity results using different sets of N genes. (A) Agglomerative hierarchical clustering results for the Top N (top 1%, 5%, 10%, 25%, and 50%) genes selected. As the number of genes in each gene set increases, the clustering sensitivity decreases with a noticeable difference starting at 5% and a substantial difference increasing beyond 10% (B) Similar to Fig. 4B, flattened representations of the cerebellum are visualized as a function of the clustering shown in (A) at each corresponding sensitivity level.
